# Books Review

**Published:** 1870-03

**Authors:** 


### Books Review.

*Sleep and its Derangements.* By WM. A. HAMMOND, M. D.

No question in human psychology is, perhaps, more mysterious, and no explanation more desired than such as regards the philosophy of sleep. A glance at the book before us is sufficient to show that it eminently possesses the quality of being interesting, and a careful examination reveals the fact that the work merits the title of scientific; it is comprehensive, yet in no degree superficial, The authors investigations as to the physiological condition of the body in sleep are decisive, and the results are worth being known by both practitioner and student. We quote some important points :

"It is important to understand clearly the difference between stupor and sleep, and it is very certain that the distinction is not always made by physicians ; yet, the causes of the two conditions have almost nothing in common, and the phenomena of each are even more distinct. 1. In the first place, stupor never occurs in the healthy individual, while sleep is a necessity of life. 2. It is easy to awaken a person from sleep, while it is often impossible to arouse him from stupor. 3. In sleep the mind may be active, in stupor it is as it were dead. 4. Pressure upon the brain, intense congestion of its vessels, the circulation of poisoned blood through its substance, cause stupor, but do not induce sleep. For the production of the latter condition a diminished supply of blood to the brain, as will be shown hereafter, is necessary.

" Perhaps no one agent so distinctly points out the difference between sleep and stupor as opium, and its several preparations, A small dose of this medicine acting as a stimulant, increases the activity of the cerebral circulation, and excites a corresponding increase in the rapidity and brilliancy of our thoughts. A larger dose lessens the amount of blood in the brain, and induces sleep. A very large dose sometimes dimlnishes the power of the whole nervous system, lessens the activity of the respiratory functions, and hence allows blood which has not been properly subjected to the influence of the oxygen of the atmosphere to circulate through the vessels of the brain. All its effects are due to its influence on the heart and blood vessels, through the medium, however, of the nervous system. This point can be made plainer by adducing the results of some experiments which I have lately performed.

*Experiment.*—I placed three dogs of about the same size under the influence of chloroform, and removed from each a portion of the upper surface of the skull an inch square. The dura mater was also removed, and the brain exposed. After the effects of the chloroform had passed off—some three hours subsequent to the operation—I administered to number one, the fourth of a grain of opium, to number two one grain, and to number three, two grains. The brain of each was at the time in a perfectly natural condition. At first the circulation of the blood in the brain was rendered more active, and the respiration became more hurried.—The blood vessels, as seen through the openings in the skulls, were fuller and redder than before the opium was given, and the brain of each animal rose through the hole in the cranium. Very soon, however, the uniformity which prevailed in these respects was destroyed. In number one the vessels remained moderately distended and florid for almost an hour, and then the brain slowly regained its ordinary appearance. In number two the active congestion passed off in less than half an hour, and was succeeded by a condition of very decided shrinking, the surface of the brain having fallen below the surface of the skull, and become pale. As these changes supervened, the animal gradually sank into a sound sleep, from which it could easily be awakened. In number three the surface of the brain became dark, almost black, from the circulation of blood containing a superabundance of carbon, and owing to a diminished action of the heart and vessels it sank below the level of the opening, showing, therefore, a diminished amount of blood in its tissue. At the same time the number of respirations per minute fell from 26 to 14, and they were much weaker than before. A condition of complete stupor was also induced from which the animal could not be aroused. It persisted for two hours. During its continuance, sensation of all kind was abolished, and the power of motion was altogether lost.

"The condition of the brain during stupor is very different from that which exists during sleep; in the one case its vessels are loaded with dark blood, in the other they are comparatively empty and the blood remains florid. I think it will be sufficiently established in the course of these remarks, that sleep is directly caused by the circulation of a less quantity of blood through the cerebral tissues than traverses them while we are awake.

"During sleep the three great divisions of the mind are differently affected. 1. Feeling, embracing sensation and emotion, is suspended, so far as the first is concerned; but it is in full action as regards the second. We do not see, hear, smell, taste or enjoy the sense of touch in sleep, although the brain may be aroused into activity and we may awake through the excitations conveyed to it by the special senses. The emotions have full play, unrestrained by the will and governed only by the imagination. 2. The Will or Volition is entirely suspended.

3. The Thought or Intellect is variously affected in its different powers. The imagination is active and the memory may be exercised to a great extent, but the judgment, perception, conception, abstraction and reason are weakened, and sometimes altogether lost."

The subjects of dreams, somnambulism, wakefulness, somnolence, etc., are considered at length, and the book cannot fail to interest all who obtain it.

---

*Annual Report of the Board of Regents of the Smithsonian Institution, for the year* 1868.

In addition to the official reports, this volume presents in the appendix, memoirs of Cuvier, Oersted, Encke and Hodgkinson; a notice also of the life and scientific attainments of Schonbein, the discoverer of Ozone, gun-cotton, etc. Papers are also published on the recent progress in, and principles of the theory of heat, on the continuous vibratory motion of all matter, ponderable and imponderable, and on radiation, by Tyndall. The efficiency of the Institution seems to be on the advance, and the number of its supporters are increasing. Prof. Henry, the Secretary, states that the Institution has received into its charge all specimens belonging to the Army Medical Museum, which properly relate to to ethnology, while in turn, it has entrusted to the charge of the Surgeon General, its large collection of Crania and also all its specimens pertaining to anatomy, physiology, medicine and surgery. Our Army Medical Museum, on the best French authority is said to be the finest of its kind in the world. The volume of the Report contains much valuable information, and will be sought for by the scientists of our country.

---

*The Physiology of Man.* By AUSTIN FLINT, JR., M. D.

This volume, the third in the series, is upon "Secretion, Excretion, Ductless Glands, Nutrition, Animal Heat, Movements, Voice and Speech," this being the completion in the three volumes now published, of all the subjects belonging to human physiology, that are usually taught in medical schools, or are treated of in systematic works, except the functions of the nervous system and the processes of generation and development. As to the prospects of the remaining volume, the author says, "he has no apology to make for the apparent delay in the issue of the present volume. His labor upon it has been almost unremitting since the issue of the volume upon Alimentation, Digestion and Absorption; and his chief endeavor has been to make it represent faithfully the existing state of the science, without sparing time or pains. All he can promise is, that the remainder of the work will be prepared with equal care, and it is hoped, within a shorter interval."

It will be remembered that some of the subjects taken up in this volume, have received from the author, special investigation, by original experiments, which have developed new facts of great value, one of the most important of which. is the discovery of an excretory function of the liver, that has never before been described, and the mechanism of glycogenesis, which seems also to be now settled. The French *Academy of Sciences* awarded the author a prize for his investigations upon the excretory functions of the liver, and after endorsement by this society, he may, very naturally and properly speak with great confidence upon the subject. This volume fully sustains the high opinions everywhere expressed of this work, which, when completed, will be found the most comprehensive, scientific and complete work upon human physiology.

---

*On the Physical Basis of Life.* By T. H. HUXLEY, L. L. D., F. R. S. CHARLES T. CHATFIELD, New Haven.

This is a discourse originally delivered in Edinburgh, Nov. 18, 1868, and was subsequently published in London as the leading article in the *Fortnightly Review* for February, 1869, attracting so much attention that five editions of that number of the Magazine have already been issued. The author is Thomas Henry Huxley, of London, Professor of Natural History, in the Royal School of Mines, and of Comparative Anatomy and Physiology, in the Royal College of Surgeons. He is also President of the Geological Society of London.

In this discourse the author attempts to show that life has a physical basis—protoplasm being the scientific name for the substance which may be regarded as the physical basis of life. He proceeds to show the relations between the plant and animal, and that both spring from "protoplasm." He says : "Traced back to its natural state, the nettle arises as the man does, in a particle of nucleated protoplasm, and in the lowest plants, as in the lowest animals, a single mass of such protoplasm may constitute the whole plant, or the protoplasm may exist without a nucleus. Under these circumstances it may well be asked, how is one mass of non-nucleated protoplasm to be distinguished from another ? Why call one "plant" and another "animal ?" The only reply is that, so far as form is concerned, plants and animals are not separable, and that, in many cases, it is a mere matter of convention, whether we call a given organism an animal or a plant."

Commencing to quote this author, we cannot find place to stop, for the whole discourse is full of thought and suggestion. It cannot, as yet, be said that the sources of life have been discovered, but it would appear as though the finger of discovery was about to point it out. This discourse is upon *the question of the times*, and every thinking mind will be most deeply interested in the perusal. It may be obtained of the publisher, Charles Chatfield, of New Haven, Conn., and we earnestly advise our readers to carefully peruse it.

---

*The History of Four Cases of Chronic Inversion of the Uterus, with an account of an Operation designed as a substitute for Amputation.* By T. GAILLARD THOMAS, M. D.

This is a monograph upon the subject of Chronic Inversion of the Uterus, giving the history of this condition from the earliest account of it, to the present time, together with the various methods of treatment which have been proposed, and the results of reported cases. He relates his cases and gives the following statement of what he would do in a case of Chronic Inversion :

"In a case presenting itself for the first time for treatment, I should use belladonna and the warm douche for a week, so as to relax the uterine tissue as far as possible, and then for another week employ pressure by means of a caoutchouc bag filled with air or water. After this I should employ taxis, for a period not exceeding one or two hours, once, or at most twice a week, in the meantime keeping up vaginal pressure by the caoutchonc bag, or, if the fundus were returned within the os, by closure of this after Emmet's method.

"Having failed with these measures, *and not before,* I should resort to abdominal section, modifying the operation which I performed in the following manner. Instead of employing a dilator of two limbs, I should employ one of four; and instead of dilating by the hand applied to the handles, I should distend the instrument by screws. Having distended its four limbs, I should keep the instrument in place for twenty-five or thirty minutes, so as to wear out the tendency to contract before any efforts at reduction were made. Even then, before removing the dilator, I should introduce between its limbs something which would exert a counter-pressure against the hand placed in the vagina."

---

*Surgery of the Cervix in connection with certain Uterine Diseases.* By THOMAS ADDIS EMMETT, M. D.

We call attention to the first statement made by the author with great pleasure. "Division of the Cervix for the relief of Dysmenorrhœa and Sterility has been a favorite practice for years past with many of the profession. Scarcely any operation in surgery, however, has been proposed, where so little judgment, as a rule, has been exercised, and where its indiscriminate practice has even amounted to malpractice. A re-action has slowly taken place in the views of the profession regarding this operation, but from one extreme we fall into the opposite error, as we have certain conditions of the Uterus, which cannot be relieved otherwise than by a division of the Cervix." We give this much of a quotation, leaving our readers to draw their own inferences. Our author proceeds to discuss the feasibility and propriety of the operation, and describe the conditions when it may be made with advantage.

Upon the question of the removal of the uterine neck, he says: Amputation of the Cervix is unnecessary except for removal of malignant disease or elongation of the neck, where the excessive growth is sometimes several inches. In conclusion, he speaks of the practice "too common" to direct treatment to the observed conditions of the neck, when in reality this is only the outcropping of the disease within the canal. The pamphlet is instructive, and practitioners will do well to read and ponder.

---

*The Physical Life of Woman: Advice to the Maiden, Wife and Mother.* By GEORGE H. NAPHEYS, A. M., M. D.

This book comprises a popular discussion of many subjects which the curious of both sexes are anxious to know about. The author has introduced a great variety of topics, and for the most part, his teachings are according to the best modern authors, the text being translated into popular style, so as to be within the comprehension of all.

We do not think that it is really desirable to instruct the ladies of our country in all these matters, but perhaps it is difficult to keep them in ignorance, and so better to have them understand the truth, than believe in error. There are no subjects connected with the virtues or sins of mankind which are not now publicly and popularly discussed and illustrated; we may fairly be said to be without secrets or privates. If as well thus as any way, then this book should be in the hands of fathers, mothers, sons and daughters.

